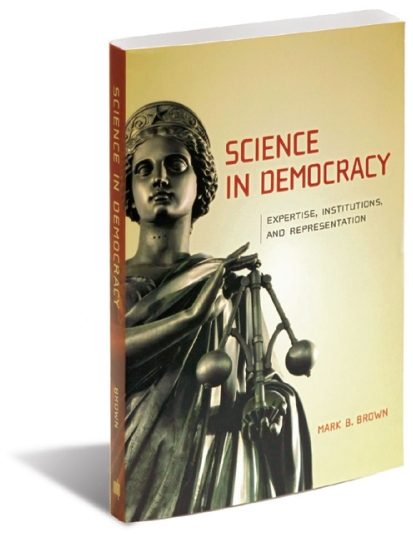# Science in Democracy: Expertise, Institutions, and Representation

**Published:** 2010-07

**Authors:** Sheila Jasanoff

**Affiliations:** Sheila Jasanoff is Pforzheimer Professor of Science and Technology Studies at the Harvard Kennedy School. She has authored more than 100 works on the role of science and technology in the law, politics, and public policy of modern democracies, with particular focus on expertise, evidence, and public reasoning

What is science’s proper role in democracy? A half-century ago the answer seemed plain: to “speak truth to power.” Science and politics existed in separate spheres of facts and values. Science took its remit directly from nature and needed to pay no dues to politics. Politics, for its part, had to take facts on board in order to make sound decisions for the good of the people. Politicians did not have to concern themselves with how science discovered facts: It was sufficient that scientific facts were reliably available when needed to clarify options or justify action.

Today, the question of science’s role in democracy is much more murky, as Mark Brown, a political theorist, observes in this timely exploration of the relationship between natural knowledge and political action. *Science in Democracy* appears at a moment when trust in institutional authority seems to be waning. Even science’s ability to set a baseline of reality that politicians dare not ignore has been called into question. These are good reasons for taking a closer look at how science functions in democracies. Brown’s title promises to do just that, but to those interested in improving the quality of science in policy, the book offers less than it promises.

The pivot for Brown’s analysis is the concept of representation. Both science and democracy function through representation, which for him means “to stand for.” Scientific representations stand for entities and phenomena in the natural world. Political representation stands for the voices of the many, with devices such as elections authorizing a few to speak legitimately for wider communities. In each case, representation produces something that mimics the “real thing” (nature or polity), but partially, imperfectly, and with inevitable distortions. Why, then, should we trust either scientific or political representation?

In answer, Brown reviews major theorists of political representation, from Machiavelli at the turn of the 16th century to the contemporary French philosopher and sociologist of science Bruno Latour. Intrinsic to all notions of political representation, Brown shows, is a more or less explicit theory of how the representative’s knowledge and expertise relate to those of the people she stands for. This opens up difficult questions: Is the representative’s job to speak for common sense or the general will? Or is it to rise above popular understandings, which may be driven by passion or ignorance, and to represent levels of wisdom and experience that the multitude cannot aspire to? The answers clearly depend on the assumptions one makes about what democratic representation means: Is it to speak for all; or for the most rational, the best informed, or the most numerous; or something entirely different?

Those are issues that mature democracies are wrestling with to this day. Brown raises, but does not satisfactorily answer, a reciprocal question that democracy theorists have rarely addressed. He follows leading scholars in the field of science and technology studies to argue that values are involved in making images of nature no less than in creating model polities. Therefore, it makes sense to ask not only whether a scientific claim bears a reasonable relation to nature, but also whether the people making the claim are the right kinds of people to speak for the phenomena they represent. Indeed, if one looks at current debates in environmental policy—for example, about climate change, geoengineering, multiple chemical sensitivity, or endangered species—disputes often center on the credibility of particular spokespersons rather than the reliability of their claims. In this respect, a core question of democracy—how good are our representatives—has infiltrated science.

There is a substantial literature on the quality and reliability of science as it relates to policy and the public good. It covers such topics as technical controversies, expert advice, peer review, regulatory standard setting, the treatment of uncertainty, and the politics of technology. Though not couched as political theory, this literature has made enormous contributions to our thinking about public participation and good decision making. In effect, these works theorize scientific and democratic representation with a richness and immediacy that one cannot find in the great political thinkers of the past.

Apart from his chapter on Latour, whose treatment of democracy lacks much empirical support, Brown essentially ignores this growing body of work. One wishes he had construed his project ambitiously enough to bring this literature into conversation with the giants of classical political theory who colonize his imagination. As it is, he has laid a cornerstone for a book that remains to be written, and that may in time occupy the same shelf as Machiavelli, Hobbes, Rousseau, and Dewey.

## Figures and Tables

**Figure:**